# Study on the Characteristics of Cement-Based Magnetoelectric Composites Using COMSOL

**DOI:** 10.3390/ma18215027

**Published:** 2025-11-04

**Authors:** Weixuan Huang, Cuijuan Pang, Jianyu Xu, Kangyang Liang, Cunying Fan, Zeyu Lu, Chuncheng Lu

**Affiliations:** 1 College of Ocean Engineering and Energy, Guangdong Ocean University, Zhanjiang 524090, China; 2 Guangdong Provincial Key Laboratory of Intelligent Equipment for South China Sea Marine Ranching, Guangdong Ocean University, Zhanjiang 524088, China; 3 School of Materials Science and Engineering, Southeast University, Nanjing 211189, China

**Keywords:** finite-element analysis, cement-based magnetoelectric composite, magnetoelectric effect, magnetoelectric coefficient

## Abstract

A multiphysics-coupled 2–2 cement-based magnetoelectric composite model is established in COMSOL 6.2. This model is used to not only systematically investigate the magnetoelectric-coupling behavior, but also quantify the effects of the magnetic field, frequency, and layer-thickness ratio on the material’s magnetoelectric properties. The results demonstrate that the model effectively reproduces the internal stress–strain distribution and voltage evolution. Specifically, the magnetostrictive and piezoelectric layers exhibit mechanical responses with pronounced non-uniformity, which is attributed to boundary effects. The bias magnetic field plays a crucial regulatory role: the output voltage increases linearly from 0 to 2000 Oe and then saturates at higher fields. Under an alternating magnetic field, the composite exhibits pronounced resonance characteristics, whose frequency is jointly governed by structural dimensions and the bias field. The dynamic response was further analyzed using the magnetic flux density modulus, displacement profiles at selected locations, and voltage evolution across the piezoelectric layer. Notably, the thickness of each functional phase exerts a pronounced and distinct influence on the composite’s magnetoelectric coupling, with markedly different trends between phases. Optimization results show that a thin piezoelectric layer combined with a thick magnetostrictive layer yields the highest magnetoelectric performance. Additionally, the longitudinal and transverse magnetoelectric coefficients exhibit markedly different coupling mechanisms—this is owing to the misalignment between the magnetic-field and electric-polarization directions, and this difference further reveals the intrinsic anisotropy of the magnetoelectric response. Overall, this study provides a crucial theoretical foundation for the design and optimization of high-performance cement-based magnetoelectric composites.

## 1. Introduction

As an important multiphysics coupling phenomenon, the magnetoelectric effect shows unique advantages in the fields of energy conversion, information storage, and sensors. The study of this effect dates back to 1894, when Pierre Curie [1] first predicted the possibility of a magnetoelectric-coupling phenomenon in some crystals based on the principle of symmetry. However, due to the limitations of the material system and characterization technology at that time, the relevant research went through a long period of exploration, and it was not until 1961 that Astrov [2] observed an intrinsic magnetoelectric effect in Cr_2_O_3_ crystals, which really served as the prelude to experimental research.

**Figure 1:**
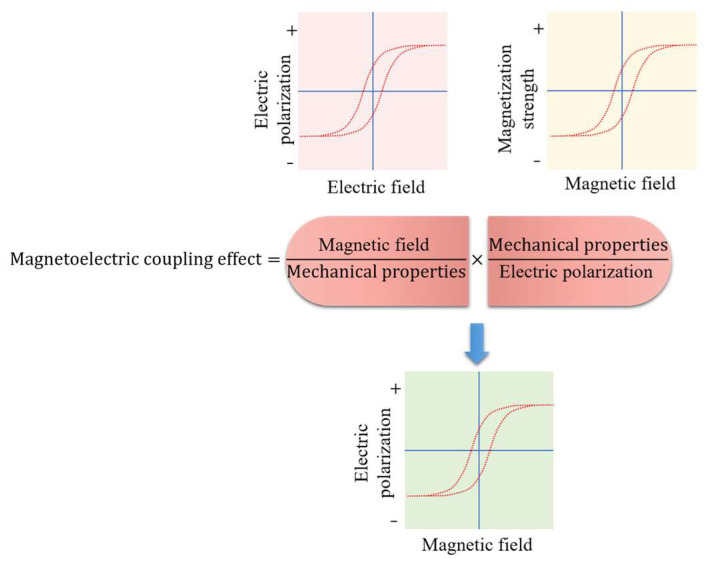
Schematic diagram of the magnetoelectric-coupling effect.

In the 1970s, with the development of composite material science, magnetoelectric research ushered in an important turning point. Suchtelen’s team at Philips Laboratory in the Netherlands (1972–1974) [3,4,5,6] pioneered the concept of composite magnetoelectric materials. Meanwhile, they constructed a CoFe_2_O_4_-BaTiO_3_ composite ceramic system [7]. For the first time, they obtained a magnetoelectric voltage coefficient of 130 mV/cm·Oe at room temperature through this system. This groundbreaking work reveals the “strain mediation” coupling mechanism: a magnetic field induces strain via the magnetostrictive effect which is transmitted by the elastic field, and this elastic field further converts the strain into an electrical signal through the piezoelectric effect. This entire process is shown in Figure 1. At that time, limitations in material preparation technologies left research in this direction stagnant, but the physical model established by this pioneering research laid an important foundation for subsequent developments.

At the turn of the century, with the rise of nanotechnology and the research on ferrous materials, magnetoelectric composites ushered in a revival. Around the year of 2000, a research group at Tsinghua University [8] proposed the theory of the giant magnetoelectric effect. Ryu’s team is from Pennsylvania State University [5], and they successfully fabricated magnetoelectric laminate composites. This achievement marked a new phase of development in this field. Nan developed the effective medium theory in 1994 [9], which is a key theoretical tool that helps us to understand the magnetoelectric coupling of composite systems.

In 2002, a Tsinghua University research group [10,11,12] introduced a third-phase polymer to develop polymer-based magnetoelectric composites with various connectivity patterns. To satisfy the demand for device integration, research focus subsequently shifted toward magnetoelectric composite thin films [13,14]. In 2004, Ramesh’s group at the University of California, Berkeley [15,16,17] fabricated laminate thin films, propelling research on multiferroic magnetoelectric composite films. In previous studies, few people have tried to use cement as the matrix material, and there were some problems with polymer-based magnetoelectric composites [18,19,20].

Magnetoelectric composites can be applied in civil engineering structures. This application requires the consideration of two aspects with regards to the parent material: compatibility and durability matching. Due to its excellent interface compatibility, cement-based sensitive composites are especially suitable for civil engineering structures, with their interface-bonding effect with concrete structures being better than that of other sensitive materials, and their key characteristics such as acoustic impedance, elastic modulus, and volume stability showing fairly consistent coordination with the main structure’s concrete materials [21,22]; they also completely eradicate the aging problem of polymer-based materials. Studies have provided evidence that cementitious piezoelectric composites have addressed two key issues—compatibility and inconsistent durability with the parent material—and are highly suitable for the low-frequency vibration environments of concrete structures, which range from 0.1 to 50 Hz. This cementitious piezoelectric composite effectively reduces the polarization voltage and improves the polarization efficiency. Under the same conditions, it also has better mechanical strength, electromechanical-coupling performance, piezoelectric performance, and reliability. Its piezoelectric strain constant d_33_ and piezoelectric voltage constant g_33_ are higher than those of corresponding polymer-based piezoelectric composites [23]. Moreover, self-sensing drives have been developed for concrete structures based on cementitious piezoelectric composites and have successfully been applied to actual engineering structures [24]. Previous studies have shown that cement particles in magnetoelectric composites have two key roles due to their inherent properties [25,26,27]. First, their cementitious properties allow them to act as binders for various functional phases. Second, their high mechanical strain–stress transmission efficiency enables them to serve as an important transport medium. Thanks to these roles, the composites have better magnetoelectric properties and sensitivity than other magnetoelectric composites. They also exhibit better versatility, including dielectric properties, piezoelectric properties, and magnetostrictive properties.

Previous experimental studies have achieved a certain degree of performance control over cementitious magnetoelectric composites under specific conditions, including structure, ratio design, and magnetoelectric-coupling voltage coefficient tests; however, more extensive performance optimization design requires establishing a theoretical model of the composites’ magnetoelectric-coupling behavior. Building on the previous focus on a 2–2 sandwich magnetoelectric composite structure, a simulation model targeting a 3D cantilever beam magnetoelectric composite structure [28,29] was constructed using the COMSOL Multiphysics finite-element analysis platform. This model breaks through the limitations of traditional 2D simulation related to geometric simplification and boundary conditions and significantly improves simulation accuracy under actual working conditions. In material modeling, the magnetostrictive phase uses a linear-elastic constitutive model coupled with the Jiles–Atherton hysteresis model (H–B curve) to characterize its nonlinear magnetization properties, while the piezoelectric phase is described by strain–charge-coupling constitutive equations to account for its electromechanical transduction behavior. This coupled modeling approach enables the structural design and parameter optimization of the three-layer laminated magnetoelectric composite.

## 2. Model Construction

**Figure 2:**
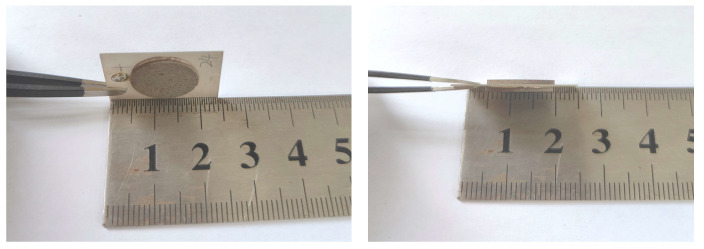
Physical sample size diagram.

The model is a type 2–2 cementitious magnetoelectric composite structure consisting of an upper and lower magnetostrictive layer and an intermediate piezoelectric layer, as shown in Figure 2 below. The magnetoelectric effect is closely related to the coupling between the upper and lower layers and the application of mechanical boundary conditions, with the interface assumed to be in an ideal coupling state. The thickness of the magnetostrictive layer is represented by the parameter *t*_m_, and that of the piezoelectric layer by another parameter *t*_p_. In the model, a uniform magnetic field is applied along the *y*-axis, the magnetization is oriented along the *y*-axis, and the piezoelectric layer is polarized along the *z*-axis.

### 2.1. Multiphysics-Coupled Constitutive Equations

In multiferroic composites, there is a coupling between the mechanical, magnetic, and electric fields, which can be described using the coupling constitutive equation [30]:

(1) $$\varepsilon_{\mathrm{ij}}=S_{\mathrm{ijkl}}\sigma_{\mathrm{kl}}+d_{\mathrm{ijkl}}E_{k}+\lambda_{\mathrm{ijkl}}H_{k}$$

(2) $$D_{i}=\kappa_{\mathrm{ij}}E_{j}+e_{\mathrm{ijk}}\sigma_{\mathrm{jk}}$$

(3) $$B_{i}=\mu_{\mathrm{ij}}H_{j}+e_{\mathrm{ijk}}\sigma_{\mathrm{jk}}$$

Here, $S_{\mathrm{ijkl}}$ denotes the elastic compliance tensor, $E_{k}$ the electric field strength, $H_{k}$ the magnetic-field strength, $D_{i}$ the electric displacement vector, $B_{i}$ the magnetic flux density, $\varepsilon_{\mathrm{ij}}$ the strain tensor, $\kappa_{\mathrm{ij}}$ the dielectric tensor, $\mu_{\mathrm{ij}}$ the permeability tensor, and $e_{\mathrm{ijk}}$ the piezomagnetic or piezoelectric tensor.

### 2.2. Equilibrium Equations

The dynamic equilibrium equations [28] for the 2–2 magnetoelectric composite are as follows:

(4) Fv=∇iσ−ρi∂2ui∂t2(i=m,p)

Here, the subscript *i* denotes the individual materials (i=m for the magnetostrictive layer and i=p for the piezoelectric layer), ρi is the mass density, ui the displacement tensor, σi the stress tensor, and $F_{v}$ the sum of all external forces.

### 2.3. Formulas for the Magnetoelectric Coefficient

The magnetoelectric coefficient $\alpha_{\mathrm{ME}}$ [31] represents the electric-field change induced per unit change in magnetic field, defined as follows:

(5) $$\alpha_{\mathrm{ME}}=\frac{\partial E_{z}}{\partial H_{y}}$$

where $E_{z}$ denotes the z-component of the electric-field strength and $H_{y}$ the y-component of the magnetic-field strength. The final derived expression is as follows:

(6) $$\alpha_{\mathrm{ME}}=\frac{d_{\mathrm{zz}}\cdot \lambda_{\mathrm{xx}}}{t_{p}}$$

The result shows that the magnetoelectric coefficient $\alpha_{\mathrm{ME}}$ is directly proportional to the piezoelectric coefficient $d_{\mathrm{zz}}$ and the magnetostrictive coefficient $\lambda_{\mathrm{xx}}$, and inversely proportional to the thickness *t*_p_ of the piezoelectric layer.

## 3. Finite-Element Analysis

A three-dimensional magnetoelectric composite structure was built in COMSOL 6.2 (COMSOL Inc., Burlington, MA, USA). As shown in Figure 2, the upper and lower layers are circular disks with a radius of 7 mm and a thickness of *tm*, whereas the intermediate layer is a rectangular block of 25 mm × 20 mm × *t*_p_ (with *t*_m_ = 1 mm and *t*_p_ = 0.6 mm). One end of the structure is fully clamped, and the opposite end remains free. The properties of Portland cement, the magnetostrictive material Terfenol-D, and the piezoelectric material PZT-5H are detailed in Table 1.

**Figure 3:**
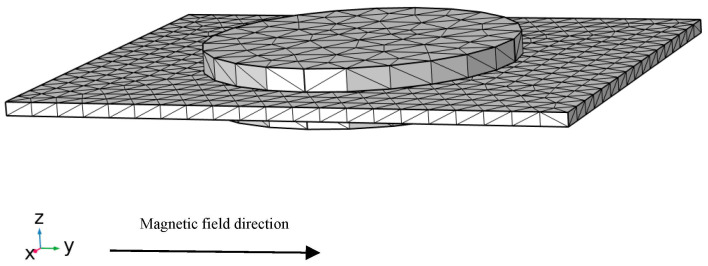
Mesh schematic of the magnetoelectric composite structure model; the uniform magnetic field is applied along the *y*-axis of the coordinate system.

The entire analysis is conducted within COMSOL’s multiphysics environment, integrating electric, magnetic, and elastic fields; their interrelationships are illustrated in Figure 1. In COMSOL, the three physical fields are fully coupled. Figure 3 presents the geometric model analyzed in COMSOL 6.2, with the mesh controlled by physics. Steady-state, transient, parametric, frequency-domain small-signal, and eigenfrequency analyses are performed to characterize the composite’s magnetoelectric response under both DC and AC magnetic fields.

## 4. Results and Discussion

### 4.1. Static Distribution Results

**Figure 4:**
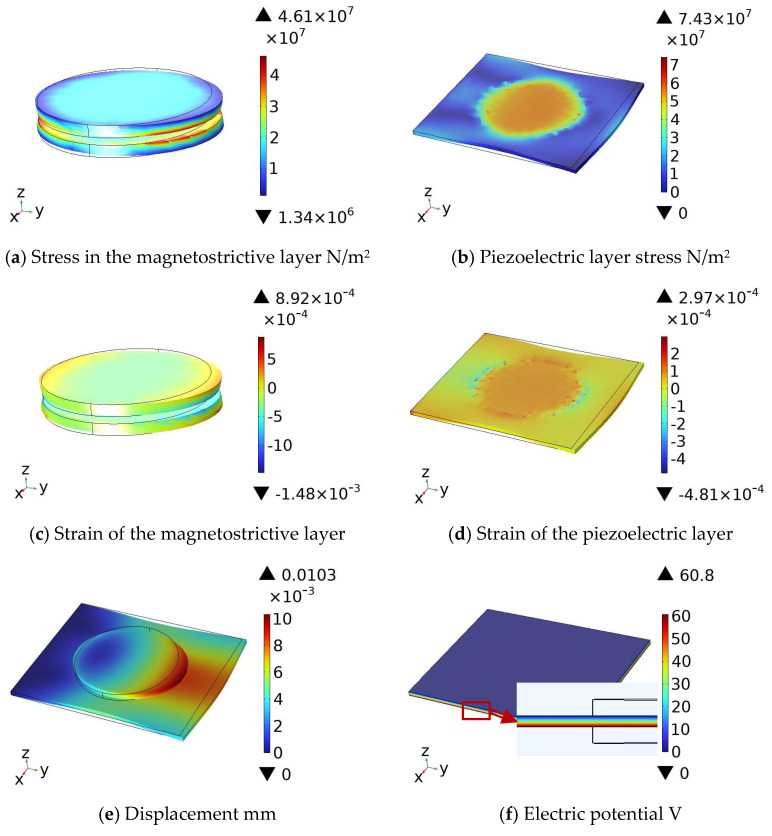
Distribution of variables in the magnetoelectric composite under a uniform DC magnetic field applied along the *y*-axis. (**a**) Stress distribution in the magnetostrictive layer; (**b**) stress distribution in the piezoelectric layer; (**c**) strain distribution in the magnetostrictive layer; (**d**) strain distribution in the piezoelectric layer; (**e**) displacement distribution; and (**f**) electric potential distribution.

Using the above approach, the geometric model was established in COMSOL and subjected to steady-state analysis. A DC magnetic field of *H*_dc_ = 10,000 Oe was applied along the *Y*-axis around the composite structure. Figure 4a–d present the distributions of stress, strain, displacement, and electric potential within the composite structure, respectively. The figures clearly reveal a pronounced non-uniformity in both stress and strain distributions, with marked disparities among different regions. Significant stress and strain gradients appear at the magnetostrictive layer’s boundaries and its interfaces with the piezoelectric layer, resulting mainly from two combined factors: the inherent non-uniformity of the magnetostrictive effect and the imposed boundary constraints.

Figure 4a,b illustrate the stress distributions in each layer of the composite structure. Figure 4a shows that the magnetostrictive layer is in tension, with relatively uniform stress in most of the central region but high-stress zones at the exterior-contacting edges and constrained bottom boundary—an outcome of edge effects. Figure 4b reveals that the piezoelectric layer is under compression imparted by the upper and lower magnetostrictive layers. The stress is uniformly distributed beneath the central disk and increases only slightly at the disk’s edges. Figure 4c,d present the strain distributions across all layers. All layers exhibit uniform strain in the central region, with slightly higher values at the edges. The strain profiles of the upper, lower, and intermediate layers are mutually symmetric, consistent with the structural design and the uniform applied magnetic field. In Figure 4c, the magnetostrictive layer exhibits high strain at its edges, particularly along the bottom boundary where a pronounced high-strain zone appears. Because the top and bottom layers are in direct contact with the external environment, their edges experience complex magnetic-field and constraint effects, resulting in stress concentration and substantial strain—clear evidence of pronounced edge effects. Figure 4d shows that the intermediate layer, enclosed by the upper and lower layers, experiences strain governed by their simultaneous deformation; consequently, strain concentrates slightly at the edges due to the magnetostrictive effect. Since one end of the piezoelectric layer is fixed and the other is free, the free end exhibits pronounced strain. Figure 4e presents the displacement distribution. The magnetostrictive layer exhibits the largest deflection, reaching 1.03 × 10^−2^ mm, because the magnetic field acts directly on this layer. In the piezoelectric layer, the free-end displacement increases with distance from the fixed end, with the gradient dictated by the material’s displacement profile. Regions of the piezoelectric layer not in contact with the central disk display a centrally symmetric displacement pattern radiating from the disk; areas outside this radiation zone show much smaller displacements. Figure 4f shows the electric-potential distribution in the piezoelectric layer (upper surface grounded). The potential exhibits a clear gradient along the thickness direction, transitioning from low (blue) at the top to high (red) at the bottom, indicating a mechanical-stress gradient that drives the potential variation.

### 4.2. Influence of the DC Bias Magnetic Field

**Figure 5:**
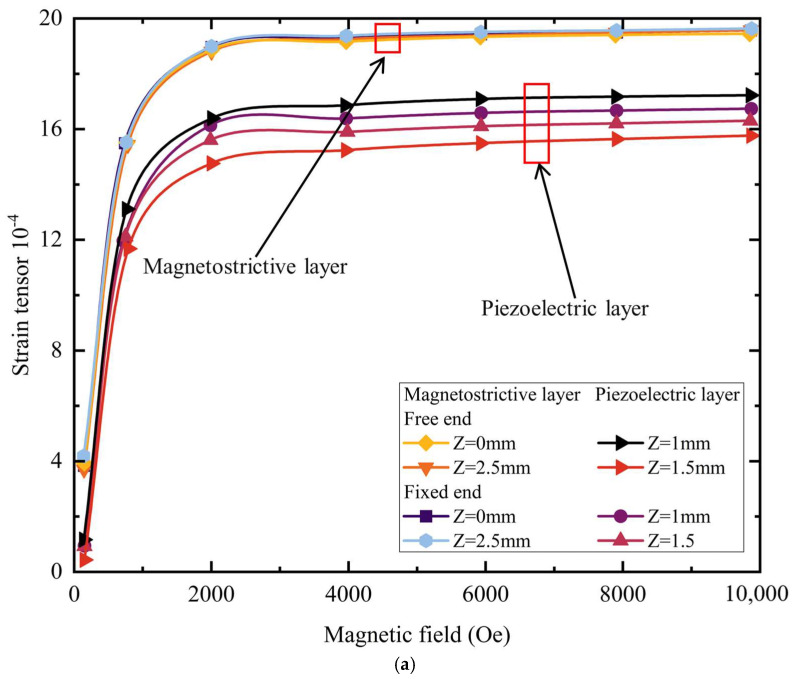

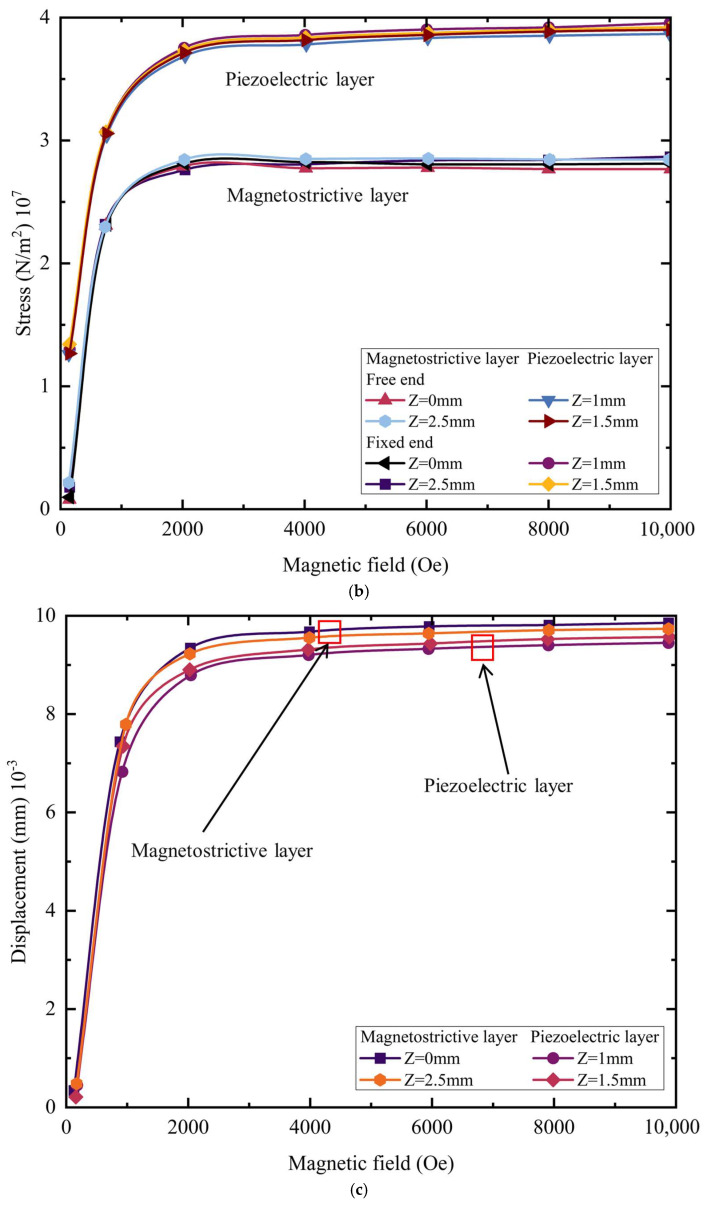

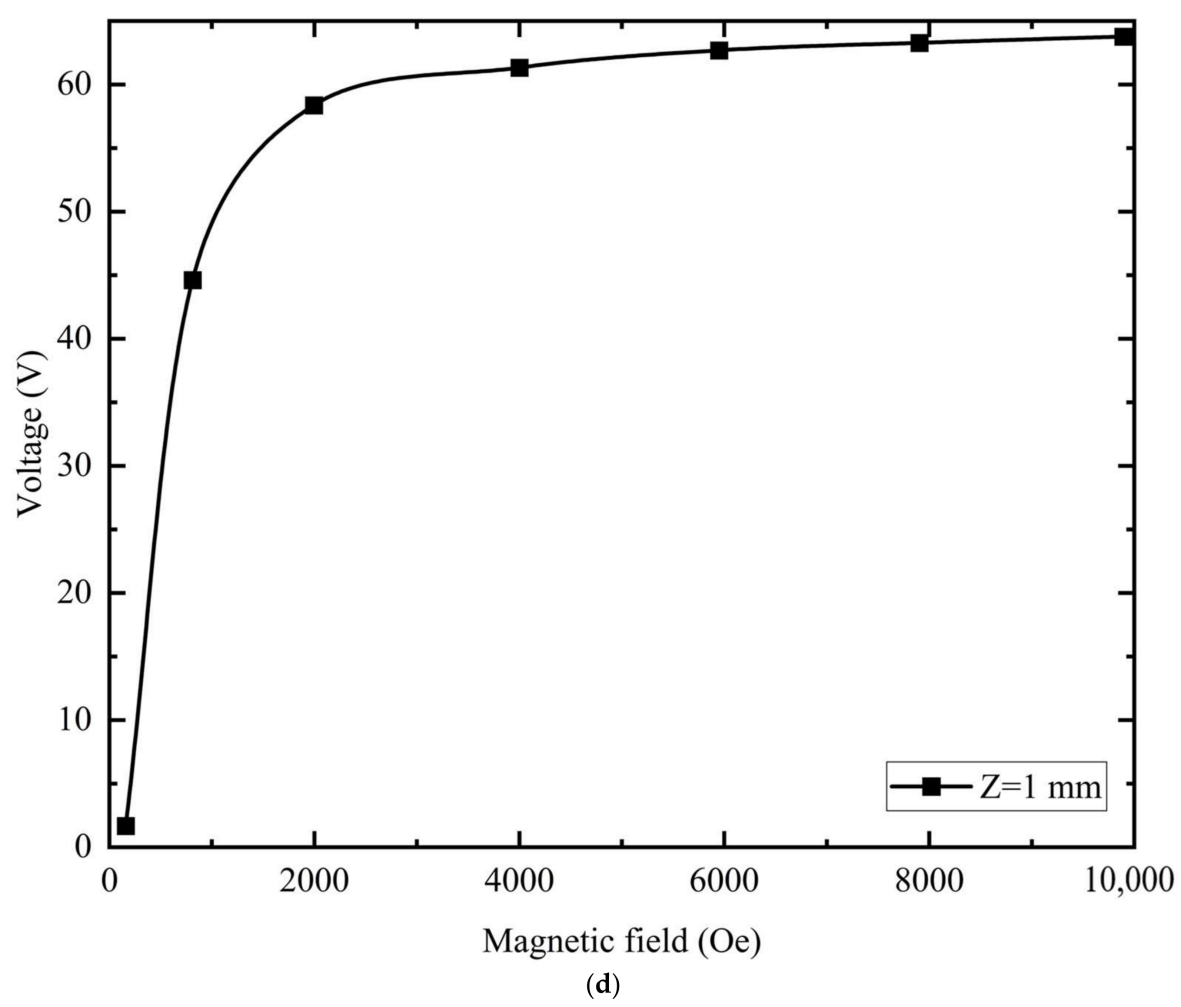
Trends of each component of the magnetoelectric composite with the magnetic field under a uniform DC field applied along the *y*-axis, and the AC magnetic field fixed at 1 Oe and the frequency at 1 kHz: (**a**) strain; (**b**) stress; (**c**) displacement; and (**d**) voltage.

The magnetoelectric-coupling coefficient is the most direct indicator of a material’s magnetoelectric response. To examine the relationship between the magnetic field and this coefficient, a parametric sweep was performed in COMSOL’s steady-state solver—this sweep varies the DC bias field *H*_dc_ from 0 to 10,000 Oe while keeping the AC field *H*_ac_ at 1 Oe and the frequency *f* at 1 kHz; the resulting trends are shown in Figure 5.

Figure 5 plots strain, stress, displacement, and voltage variations with the magnetic field at different positions along the structure: labels *Z* = 0 and 2.5 denote the magnetostrictive layers, while *Z* = 1 and 1.5 correspond to the piezoelectric layer. As shown, the composite’s stress, strain, displacement, and voltage change similarly with the DC bias magnetic field—rising rapidly between 0 and 2000 Oe and tending to saturate after 2000 Oe. Figure 5a shows the strain response: under the bias field, the magnetostrictive layer first develops strain, which is then transferred to the piezoelectric layer, so the piezoelectric layer exhibits slightly smaller strain than the magnetostrictive layer. Because one end of the piezoelectric layer is fixed, the strains at its two ends differ: the maximum strain at the fixed end is about 6.45 × 10^−4^, whereas that at the free end reaches approximately 7.82 × 10^−4^. This difference arises from mechanical constraints and model geometry, which allow the free end to deform slightly more. Figure 5b presents that the piezoelectric layer experiences higher stress than the magnetostrictive layers. This is because the two magnetostrictive layers exert Z-direction forces compressing the piezoelectric layer, and the piezoelectric material’s higher elastic modulus generates greater stress under equivalent strain. Figure 5c shows that despite the higher stress on the piezoelectric layer from laminated compression by the adjacent layers, its displacement remains comparatively small: the maximum displacement in the piezoelectric layer is 8.5 × 10^−3^ mm, versus 1.03 × 10^−2^ mm in the magnetostrictive layer. Figure 5d plots the piezoelectric layer voltage versus the magnetic field. From 0 to 2000 Oe the potential rises sharply with increasing field; beyond 2000 Oe the curve flattens, reaching a maximum of approximately 60.8 V.

### 4.3. Influence of AC-Driven Magnetic Field

**Figure 6:**
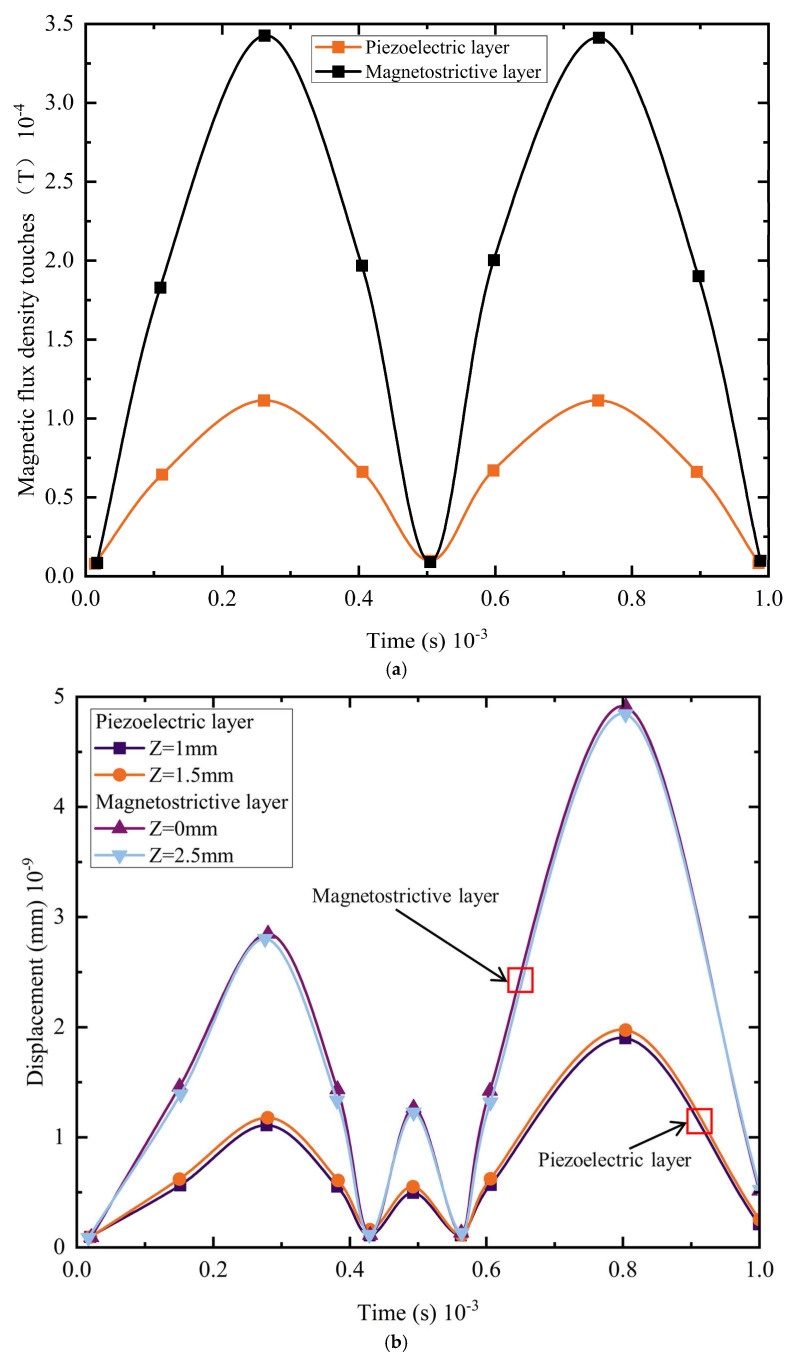

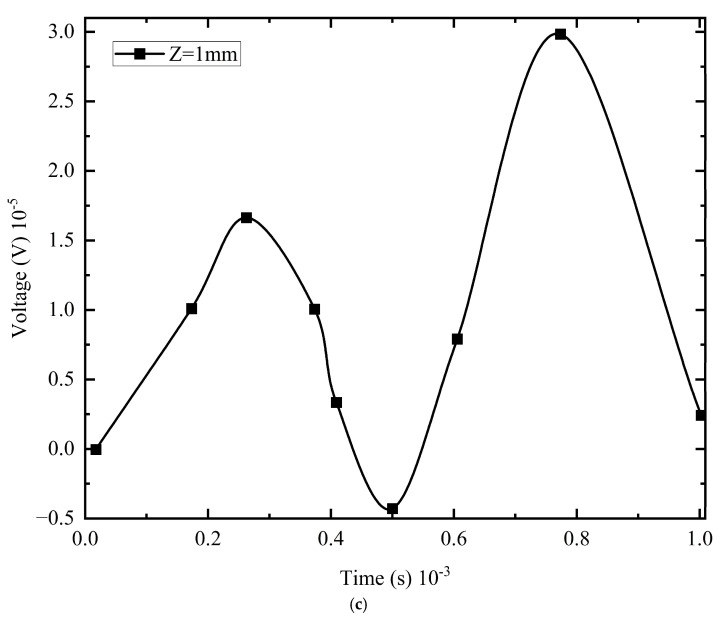
Time-dependent trends of magnetoelectric composite components under an AC field of 1 Oe applied along the *y*-axis at 1 kHz: (**a**) magnetic flux density in the magnetostrictive and piezoelectric layers; (**b**) free-end displacement; and (**c**) voltage across the piezoelectric layer.

To analyze the dynamic response, an alternating magnetic field with *H*_ac_ = 1 Oe and an applied frequency of *f* = 1 kHz is exerted on the magnetoelectric composite along the Y-direction, and variations in all relevant quantities within the composite structure are examined—the results are presented in Figure 6, where *Z* = 0 and 2.5 mm correspond to the magnetostrictive layers and *Z* = 1 and 1.5 mm denote the piezoelectric layer.

Figure 6a displays the temporal evolution of the magnetic flux density modulus in the magnetostrictive and piezoelectric layers. Both layers exhibit similar periodic variations, but the magnetostrictive layer—a ferromagnetic material—shows pronounced magnetization, with its peak flux density reaching 3.47 × 10^−4^ T. In contrast, the piezoelectric layer, which is non-magnetic, has a much weaker response: its flux density fluctuates modestly, with a lower peak of 1.07 × 10^−4^ T. This contrast highlights the distinct dynamic behaviors of the two materials under the alternating magnetic field. Figure 6b shows the displacement curves at different positions along the free end of the composite under the alternating magnetic field. Displacements vary with geometry: the magnetostrictive layer reaches a maximum of about 4.71 × 10^−9^ mm, while the piezoelectric layer attains 1.82 × 10^−9^ mm. The larger displacement in the magnetostrictive layer is consistent with the explanation given earlier. Figure 6c shows the voltage variation in the piezoelectric layer; the output voltage also follows a sinusoidal waveform, reaching a maximum of 2.99 × 10^−5^ V.

**Figure 7:**
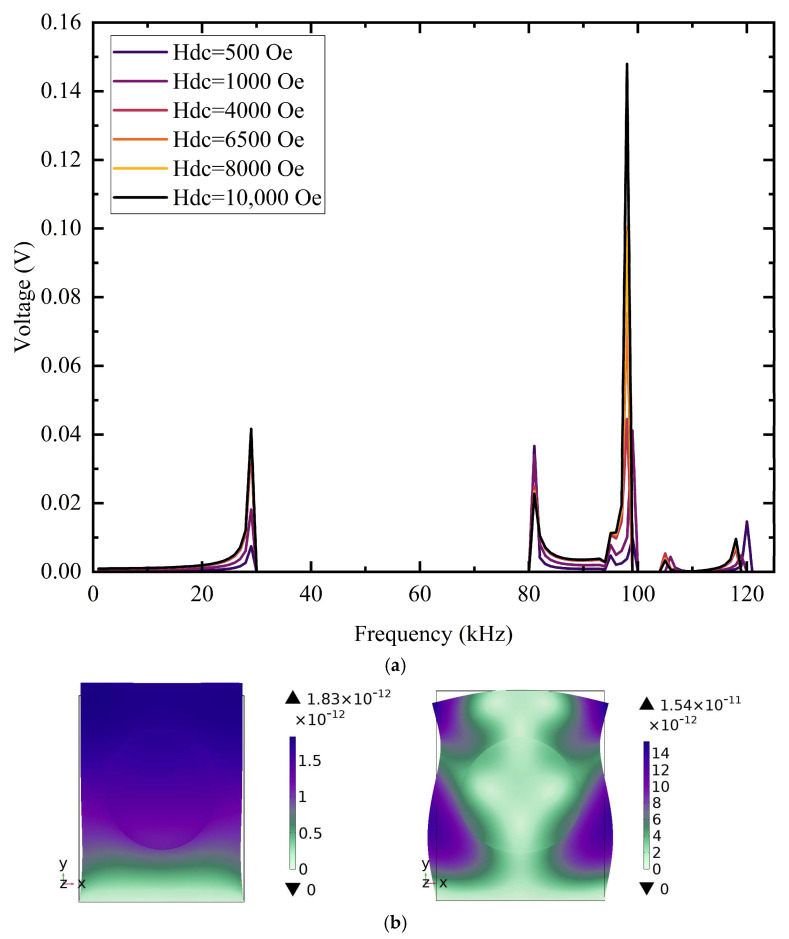
Frequency response of the composite under a DC bias field applied along the *y*-axis, with the AC field fixed at 1 Oe: (**a**) voltage versus frequency under different magnetic fields and (**b**) vibration modes.

Under an alternating magnetic field, the material exhibits pronounced resonant behavior, with structural dimensions, volume fractions of the constituent phases, and field orientation all exerting significant influence on the resonant frequency. The resonant frequency of the magnetoelectric composite is investigated via COMSOL’s small-signal frequency-domain solver—the structural dimensions of the previously defined model are retained, and a DC bias field (*H*_dc_ = 10,000 Oe) and an AC field (*H*_ac_ = 1 Oe) are imposed. Guided by prior experimental experience, only the strongest resonance peak is targeted, with the frequency swept from 1 kHz to 125 kHz; the results are presented in Figure 7.

In this study, the magnetoelectric response of the composite was examined by systematically varying the DC bias field *H*_dc_, which allowed the observation of the output-voltage characteristics. With *H*_ac_ fixed at 1 Oe, tests were carried out at *H*_dc_ = 500, 1000, 4000, 6500, 8000, and 10,000 Oe. Figure 7a shows that the composite structure attains its peak output voltage at the third-order resonance frequency of 98 kHz, reaching approximately 0.148 V—a value significantly higher than the ~0.04 V observed at other resonant modes. When the bias field is varied, the output voltage rises sharply with *H*_dc_: 0.148 V at 10,000 Oe versus 0.037 V at 500 Oe. Thus, increasing the DC bias field multiplies the resonant voltage. Figure 7b presents the eigenfrequency search with a shift around the characteristic frequencies, for which six eigenmodes were requested. It also shows the strain distributions of the first- and third-order modes at *H*_dc_ = 10,000 Oe: at the first-order resonance, the piezoelectric layer strain is 1.83 × 10^−12^, while at the third-order resonance, it rises to 1.54 × 10^−11^. The third-order strain is nearly an order of magnitude larger, consistent with the much higher output voltage observed at this resonance.

### 4.4. Influence of Different Functional-Phase-Layer Thicknesses

The relative thickness ratio of each functional phase in the magnetoelectric composite matters significantly, as it governs three key aspects—inter-layer vibration transfer, resonant behavior, and overall magnetoelectric performance—and is crucial for achieving a high coupling coefficient. A systematic investigation of the influence of functional-phase thickness is therefore necessary. To investigate the piezoelectric layer’s influence on the magnetoelectric (ME) coefficient, the $\alpha_{\mathrm{ME}}$ coefficient is calculated from Equation (5) using the average voltage on the piezoelectric layer’s upper surface. The DC magnetic field *H*_dc_ varies from 0 to 10,000 Oe, with *H*_ac_ fixed at 1 Oe and frequency *f* at 1 kHz; the magnetostrictive layer’s thickness (*t*_m_) remains unchanged at 1 mm, while the piezoelectric layer’s thickness (*t*_p_) is adjusted by a gradient from 0.35 to 2 mm. The trend of the magnetoelectric coefficient with magnetic field is then observed for different *t*_p_ values.

**Figure 8:**
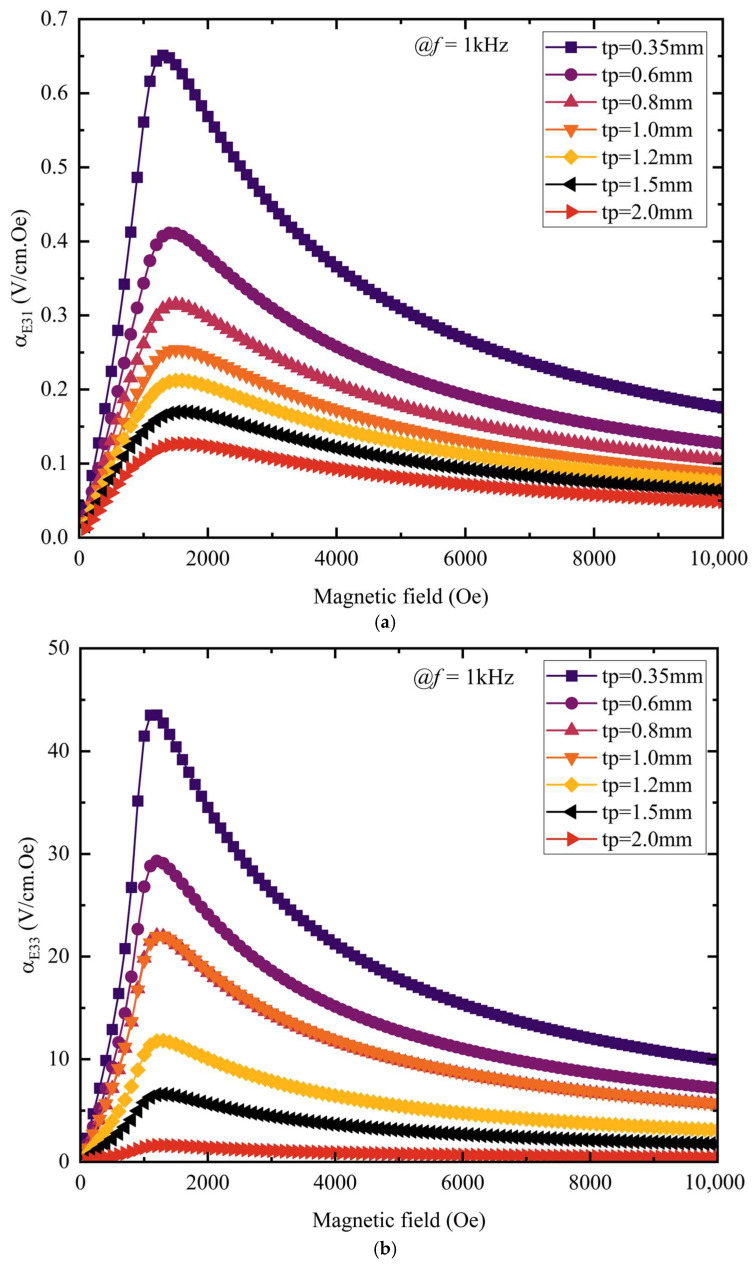
By varying the piezoelectric-layer thickness *t*_p_, we examine how the magnetoelectric coefficient evolves with the magnetic field for different *t*_p_ values: (**a**) set the magnetic field along the *y*-axis piezoelectric layer $\alpha_{E31}$ and (**b**) set the magnetic field along the *z*-axis piezoelectric layer $\alpha_{E33}$.

As shown in Figure 8a, composites of all thicknesses exhibit a consistent trend: in the low-field region below 1500 Oe, the transverse magnetoelectric coefficient $\alpha_{E31}$ increases almost linearly with *H*_dc_; thereafter, it gradually decreases as field strength further increases, exhibiting a single peak at approximately 1500 Oe. As the piezoelectric-layer thickness *t*_p_ increases, this peak shifts toward lower magnetic fields. The peak magnetoelectric coefficient of the thinner piezoelectric layer (e.g., 0.35 mm) is the largest, reaching 0.65 V/Oe·cm, and the peak curve decreases as *t*_p_ increases. This indicates that a thicker piezoelectric layer reaches its peak more easily at lower magnetic field strength, but results in a weaker magnetoelectric-coupling effect—a trend consistent with previous experimental results [26]. Figure 8b shows that the longitudinal magnetoelectric coefficient $\alpha_{E33}$ follows a trend similar to that of $\alpha_{E31}$, but attains markedly higher values; for a 0.35 mm piezoelectric layer, $\alpha_{E33}$ reaches 45.5 V/Oe·cm. This outcome stems from the differing deformation modes and vibration patterns induced by the distinct orientations of the magnetic field.

**Figure 9:**
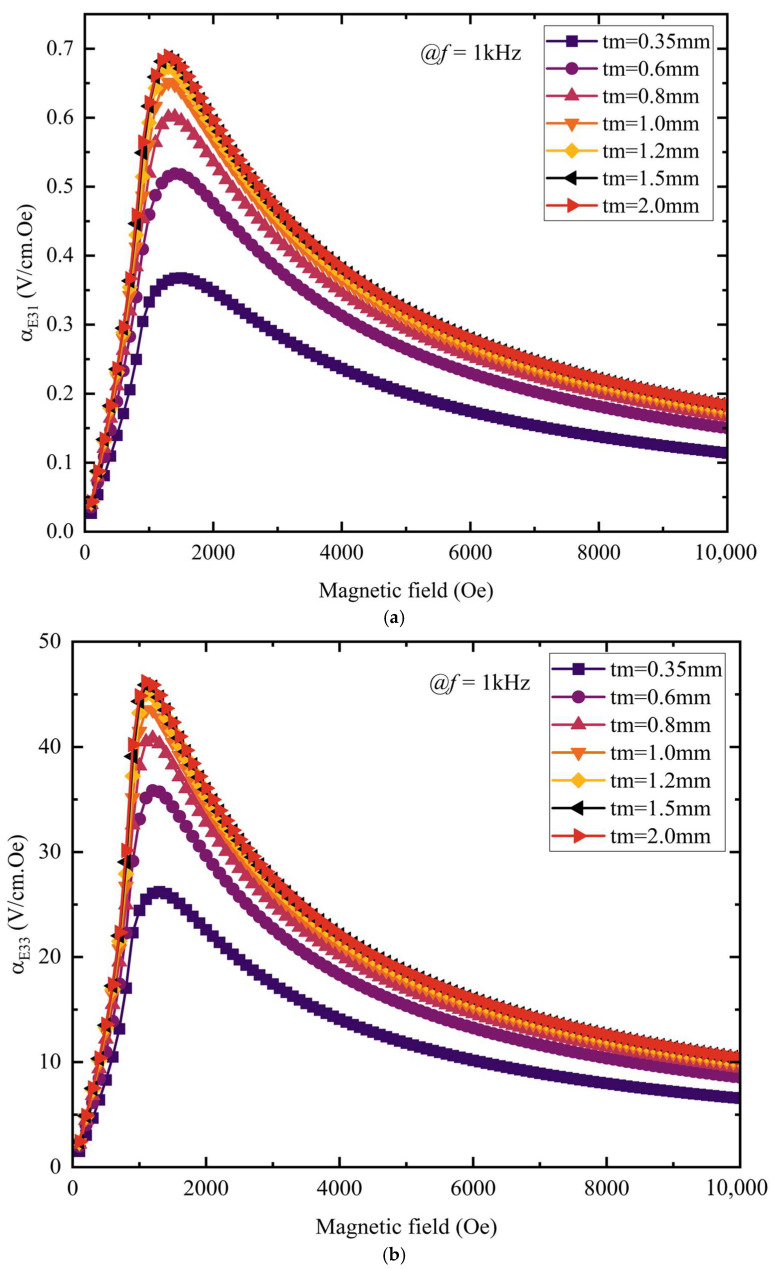
Varying the magnetostrictive-layer thickness tm, we track how the magnetoelectric coefficient changes with the magnetic field for each *t*_m_ value: (**a**) set the magnetic field along the *y*-axis magnetostrictive layer $\alpha_{E31}$ and (**b**) set the magnetic field along the *z*-axis magnetostrictive layer $\alpha_{E33}$.

Under the same magnetic field and frequency conditions, the piezoelectric-layer thickness was fixed at 0.35 mm, while the magnetostrictive-layer thickness *t*_m_ was varied stepwise from 0.35 mm to 2 mm; the resulting dependence of the magnetoelectric coefficient on the magnetic field was recorded for each *t*_m_. As shown in Figure 9a,b, the overall trend of the magnetostrictive layer’s magnetoelectric coefficient curve is roughly similar to that of the piezoelectric layer. With increasing magnetostrictive-layer thickness, the peak variation of the magnetoelectric coefficient shows obvious phased characteristics: for thicknesses between 0.35 mm and 1.0 mm, the peak magnetoelectric coefficient rises sharply with increasing thickness, indicating a rapid enhancement of the material’s magnetic-field response; beyond 1.0 mm, the peak continues to climb but at a markedly slower rate, and maxima are reached at *t*_m_ = 2 mm—with the transverse coefficient $\alpha_{E31}$ attaining 0.69 V/Oe·cm and the longitudinal coefficient $\alpha_{E33}$ reaching 46.2 V/Oe·cm. This is because the magnetostrictive layer has a critical thickness; beyond this thickness, the material’s internal magnetic-coupling effect and magnetic flux distribution become relatively stable, so further increasing the thickness has a gradually weaker effect on the peak. The foregoing results indicate that increasing the magnetostrictive-layer thickness or decreasing the piezoelectric-layer thickness enhances the composite’s magnetoelectric coefficient. This is because the strain in the piezoelectric layer is induced by the magnetostrictive layer’s deformation: when the magnetostrictive thickness is fixed, raising the piezoelectric thickness attenuates the transferred strain, lowering the output voltage and thus the magnetoelectric coefficient; conversely, with the piezoelectric thickness held constant, increasing the magnetostrictive thickness strengthens the induced strain, boosting the output voltage and thereby the magnetoelectric coefficient.

**Figure 10:**
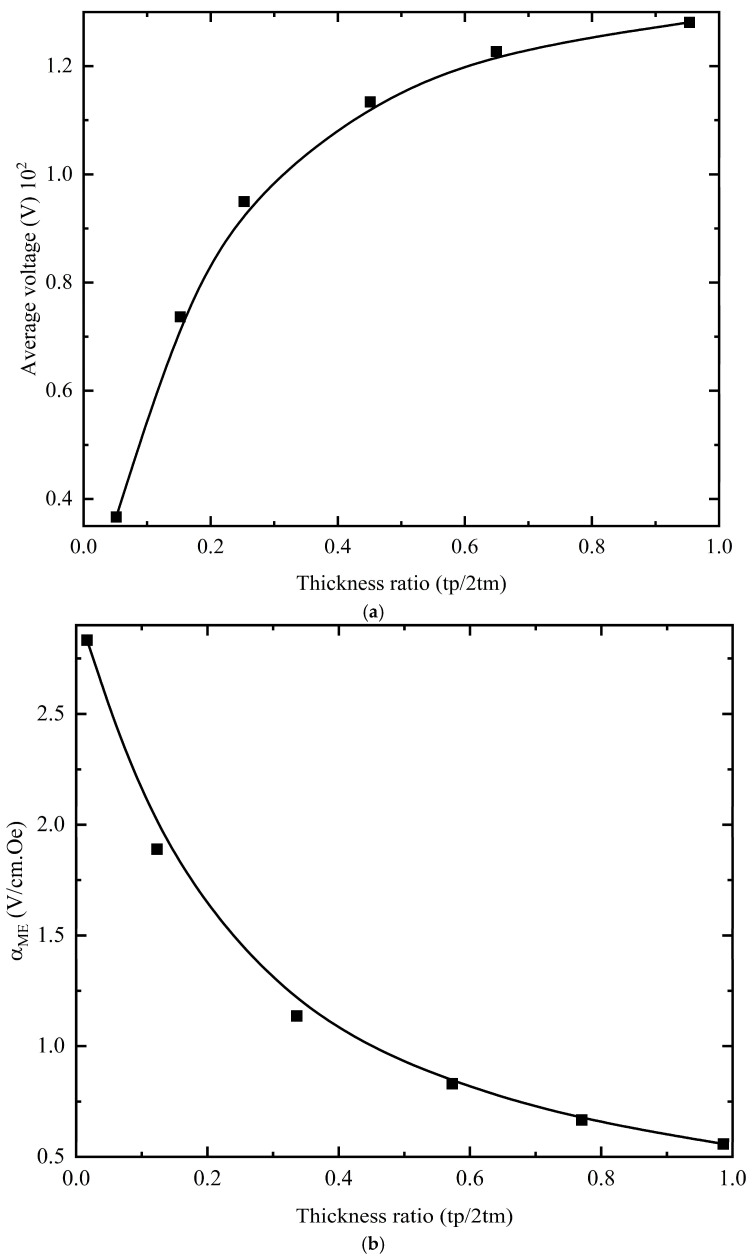
A magnetic field is applied along the *y*-axis to analyze how the *t*_p_/2*t*_m_ thickness ratio affects the magnetoelectric coefficient of the composite: (**a**) average voltage and (**b**) magnetoelectric coefficient versus thickness ratio.

Further analysis of the functional phases’ relative thickness ratio on the magnetoelectric coefficient is presented below. At *H*_dc_ = 10,000 Oe, *H*_ac_ = 1 Oe, and *f* = 1 kHz, the influence of the thickness ratio *t*_p_/2*t*_m_ was examined—the piezoelectric-layer thickness *t*_p_ was varied from 0.1 mm to 2 mm, while *t*_m_ (magnetostrictive-layer thickness) remained at 1 mm and the piezoelectric layer dimensions were fixed at 25 mm × 20 mm. Figure 10a plots the average voltage versus the thickness ratio: the voltage rises rapidly at first, then levels off. With magnetostrictive thickness constant, increasing piezoelectric thickness to enlarge the ratio significantly boosts voltage; however, once the ratio exceeds a certain value, the gain diminishes. This occurs because the piezoelectric layer’s average surface voltage depends on its total surface charge—a thicker layer can generate more charge, but greater thickness weakens inter-layer-coupled vibration, which reduces the final charge output and thus lowers the surface voltage. Figure 10b plots the magnetoelectric coefficient $\alpha_{\mathrm{ME}}$ versus the thickness ratio: as the ratio increases, $\alpha_{\mathrm{ME}}$ decreases monotonically from 2.8 V/cm·Oe to 0.6 V/cm·Oe, corroborating the preceding analysis.

## 5. Conclusions

Building on previous experimental research, this paper uses COMSOL 6.2 to construct a multiphysics coupling model for 2–2 cement-based magnetoelectric composites and establishes a theoretical model of the composites’ magnetoelectric-coupling behavior. The model theoretically simulates stress–strain, displacement distribution, and polarization in the multiphysics system. It also analyzes how environmental parameters (e.g., magnetic-field strength and frequency) and different functional-phase thickness ratios affect the magnetoelectric-coupling coefficient. The main conclusions are as follows:

(1) The developed 2–2 cement-based magnetoelectric composite multiphysics model accurately reproduces the internal stress–strain distributions, displacements, and voltage evolution. Results reveal pronounced non-uniformity in the stress and strain fields of the magnetostrictive and piezoelectric layers, which is induced by structural geometry and boundary effects.
(2) The bias magnetic field exerts a pronounced influence on the output voltage of the cement-based magnetoelectric composite, evidencing strong strain–stress–voltage coupling: strain generated in the magnetostrictive layer is transferred across the interface, inducing stress in the piezoelectric layer (further intensified by double-layer compression) and corresponding displacement. The output voltage rises linearly with *H*_dc_ in the range of 0–2000 Oe (peaking at 60.8 V) and tends to saturate when *H*_dc_ exceeds 2000 Oe—this confirms the existence of an optimal bias-magnetic-field interval.
(3) Under an alternating magnetic field, the magnetostrictive layer exhibits pronounced magnetization and larger displacements, while the piezoelectric layer’s voltage varies sinusoidally. The composite displays clear resonant behavior, whose frequency—governed by the bias field and structural dimensions—enables maximum output at a specific frequency. Elevating the bias field markedly amplifies the output voltage at the third-order resonance, with a simultaneous increase in the piezoelectric layer’s strain.
(4) The thickness ratios of the functional phases strongly influence the composite’s performance, with differing trends between the phases: increasing the piezoelectric thickness lowers strain-transfer efficiency, thus reducing the magnetoelectric coefficient, whereas thickening the magnetostrictive layer markedly enhances the coefficient (though the gain levels off beyond 1 mm). Thickness-ratio optimization reveals an optimal layer-thickness pairing for efficient magnetoelectric conversion: a thin piezoelectric layer (0.35 mm) combined with a thick magnetostrictive layer (2 mm) yields the highest magnetoelectric coefficient.
(5) Analysis of the thickness ratio’s influence on the magnetoelectric-coupling coefficient reveals that the longitudinal and transverse coefficients follow roughly the same trend but exhibit different coupling mechanisms and a significant numerical difference—this indicates that magnetic-field direction and polarization direction affect the coupling effect. Additionally, the geometric structure combination between different layers and the form of inter-layer contact also influence the coupling effect. Future work will consider further simulation studies on the effects of magnetic-field direction, composite structural design, and inter-layer contact.

## Figures and Tables

**Table 1:** Basic properties of phase materials.

Parameter	PZT	Terfenol-D	Cememt Paste
Density, ρ (kg/m^3^)	7.45 × 10^3^	2.07 × 10^3^	2.00 × 10^3^
Electrical conductivity, σ (S/m)	1.56 × 10^−4^	5.13 × 10^−6^	-
Relative permittivity, $\varepsilon_{r}$	{1704.4, 1704.4, 1433.6}	2.12 × 10^10^	-
Young’s modulus, E (Pa)	7.14 × 10^10^	6.37 × 10^10^	-
Poisson’s ratio, V	0.33	1.78 × 10^−1^	-
Saturation magnetization, $M_{s}\;$ (A/m)	-	3.95 × 10^5^	-
Initial magnetic susceptibility, χ	-	2.23	-
Saturation magnetostriction coefficient, $\lambda_{s}$	-	1.85 × 10^−3^	-
Domain coupling	-	0	-
Relative permeability, $\mu_{r}$	1	-	-
Dielectric constant, $\varepsilon_{r}$ (at 1 kHz)	-	-	56
Elastic compliance, *s*_33_ (10^−12^ m^2^/N)	-	-	72
Acoustic velocity, *V* (10^3^ m/s)	-	-	2.64
Acoustic impedance, *Z* = ρv (10^6^ kg/m^2^s)	-	-	5.3
Resistivity, ρ (Ohm·m)	-	-	20

## Data Availability

The original contributions presented in this study are included in the article. Further inquiries can be directed to the corresponding author.
